# Down-regulation of miR-129-5p via the Twist1-Snail feedback loop stimulates the epithelial-mesenchymal transition and is associated with poor prognosis in breast cancer

**DOI:** 10.18632/oncotarget.5406

**Published:** 2015-10-07

**Authors:** Yue Yu, Ying Zhao, Xiao-Hu Sun, Jie Ge, Bin Zhang, Xin Wang, Xu-Chen Cao

**Affiliations:** ^1^ The First Department of Breast Cancer, Tianjin Medical University Cancer Institute and Hospital, National Clinical Research Center for Cancer, Tianjin 300060, China; ^2^ Key Laboratory of Cancer Prevention and Therapy, Tianjin 300060, China; ^3^ Key Laboratory of Breast Cancer Prevention and Therapy, Tianjin Medical University, Ministry of Education, Tianjin 300060, China

**Keywords:** miR-129-5p, Twist1, Snail, EMT, breast cancer progression

## Abstract

The epithelial to mesenchymal transition (EMT) plays a pivotal role in breast cancer progression. We found that overexpression of miR-129-5p reversed EMT, whereas depletion of miR-129-5p induced EMT in breast cancer cells. We demonstrated that Twist1 is a direct target of miR-129-5p. Both Twist1 and Snail transcriptionally suppressed miR-129-5p expression. Levels of miR-129-5p were low in breast cancer tissues. miR-129-5p down-regulation correlated with advanced clinical stage and poor prognosis in patients with breast cancer. miR-129-5p expression negatively correlated with Twist1 and Snail expression. Thus, miR-129-5p down-regulation fosters EMT in breast cancer by increasing Twist1-Snail and activating a negative feedback loop.

## INTRODUCTION

miRNAs have been shown to alter cancer cell proliferation, invasion, angiogenesis, and the epithelial to mesenchymal transition (EMT) [1, 2], which plays an important role during malignant tumor progression and metastasis. miR-129-1 and miR-129-2, located at chromosome 7q32 and 11p11.2, respectively, produce the same 5′ prime product, miR-129-5p [3]. miR-129-5p expression is down-regulated in gastric cancer [4–6], bladder cancer [7], hepatocellular carcinoma [8, 9], medullary thyroid carcinoma [10], non-small cell lung cancer [11], glioma [12], and colorectal cancer [13]. miR-129-5p promotes apoptosis and enhances chemosensitivity in colorectal cancer, while decreased miR-129-5p expression, as a result of hypermethylation of the miR-129 promoter, is associated with poor clinicopathological factors, such as clinical stage and progression [14], in several cancers [8, 12, 13, 15–19]. However, miR-1295p expression is increased in some cancer tissue [20] and down-regulation of miR-129-5p inhibits growth and induces apoptosis in laryngeal squamous cell carcinoma [21], suggesting that miR-129-5p plays diverse roles in different cancer types.

While miR-129-5p has been studied in a variety of human cancers, its role in breast cancer is unclear as are the molecular mechanisms underlying miR-129-5p regulation. Breast cancer is one of the most common malignant tumors in females [22], and it is a heterogeneous disease with respect to molecular alteration, cellular composition, and clinical outcome [23]. The incomplete understanding of its carcinogenic mechanisms has lead to difficulties in selecting targeted treatments, contributing to low survival rates. In this study, we investigated the mechanisms of miR-129-5p regulation in breast cancer cells. We find that miR-129-5p serves as a tumor-suppressor in the development and progression of breast cancer.

## RESULTS

### miR-129-5p is a tumor suppressor in breast cancer

**Figure 1:**
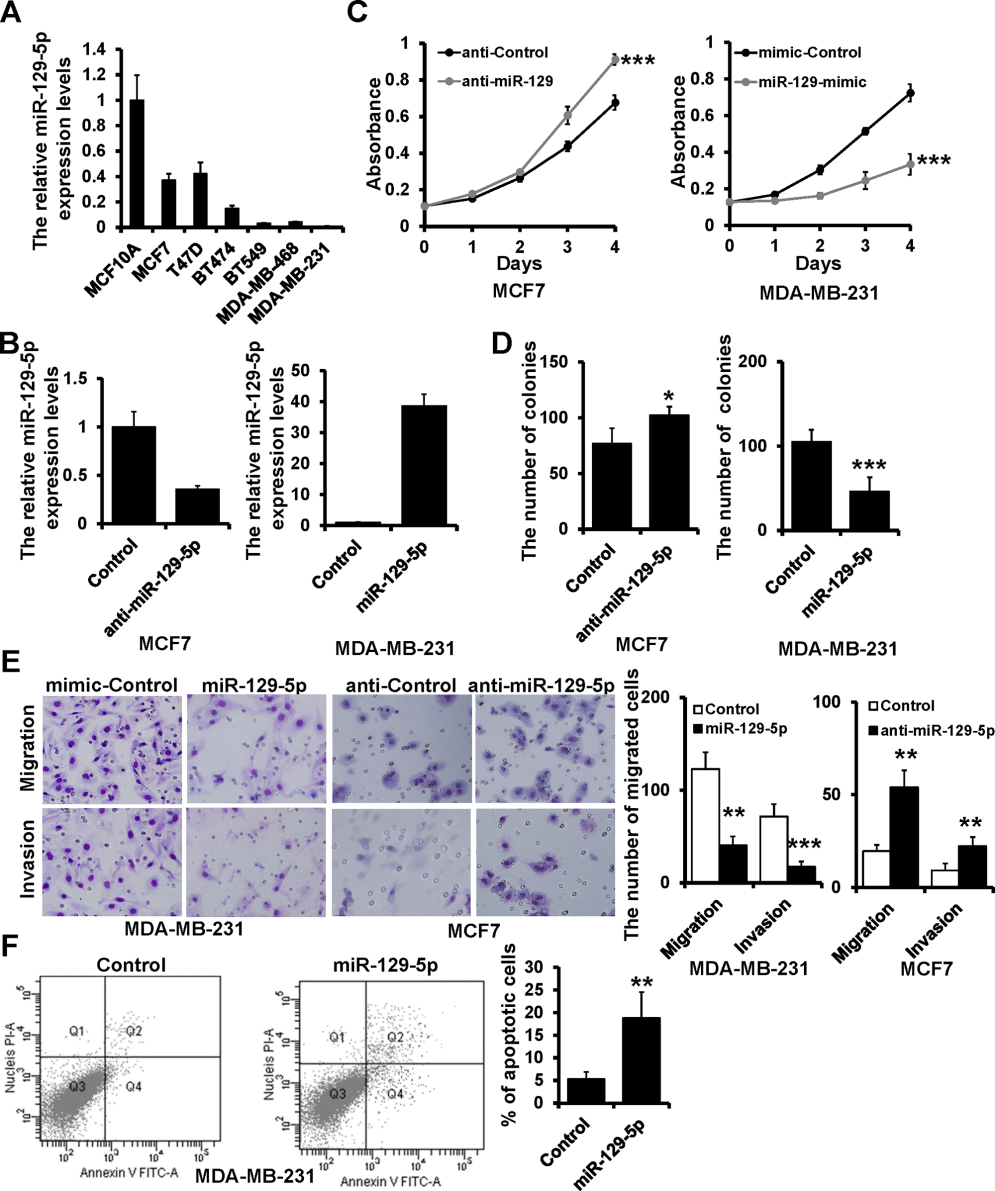
miR-129-5p suppresses breast cancer cell proliferation, migration and invasion **A.** miR-129-5p expression in the indicated breast cancer cell lines with respect to the expression in breast epithelial cell line MCF10A. **B.** RT-qPCR analysis of miR-129-5p expression in MCF7 (left) and MDA-MB-231 (right) cells transfected with the miR-129-5p inhibitor (anti-miR-129), miR-129-5p mimic (miR-129-mimic), or appropriate controls (anti-Control or mimic-Control). **C.** MTT analysis of cell growth in MCF7 and MDA-MB-231 cells treated as in B. **D.** Colony formation of MCF7 and MDA-MB-231 cells treated as in B. **E.** Representative images of migration and invasion assays in MCF7 and MDA-MB-231 cells treated as in B. Graphical representation of migrated cells. **F.** The percentage of apoptotic cells in the miR-129-5p-transfected and control MDA-MB-231 cells. (***P < 0.001, **P < 0.01, *P < 0.05).

Using RT-qPCR, we examined miR-129-5p expression in six breast cancer cell lines (MCF7, T47D, BT474, BT549, MDA-MB-468, and MDA-MB-231) and one breast epithelial cell line (MCF10A). miR-129-5p expression was down-regulated in all breast cancer cell lines as compared to MCF10A cells (Figure 1A). Next, we investigated the influence of miR-129-5p on cell proliferation by transfecting miR-129-5p inhibitor or mimic in MCF7 and MDA-MB-231 cell lines (Figure 1B). miR-129-5p inhibition resulted in a higher proliferation rate in MCF7 cells as assessed by MTT and plate colony formation assays (Figure 1C left and 1D left). In contrast, miR-129-5p over-expression reduced cell growth and produced smaller plate colonies in MDA-MB-231 cells (Figure 1C right and Figure 1D right). To investigate the role of miR-129-5p in cell migration and invasion we used a trans-well assay. miR-129-5p over-expression reduced both cell migration and invasion in MDA-MB-231 cells, while its inhibition enhanced cell migration and invasion as compared to control cells (Figure 1E). The number of apoptotic cells was significantly higher in miR-129-5p-overexpressed MDA-MB-231 cells compared with the control cells (Figure 1F). These results indicate that miR-129-5p inhibits breast cancer cell proliferation, migration, and invasion and promotes cell apoptosis, suggesting that miR-129-5p is a tumor suppressor in breast cancer.

### Down-regulation of miR-129-5p is necessary for TGF-β-induced EMT

**Figure 2:**
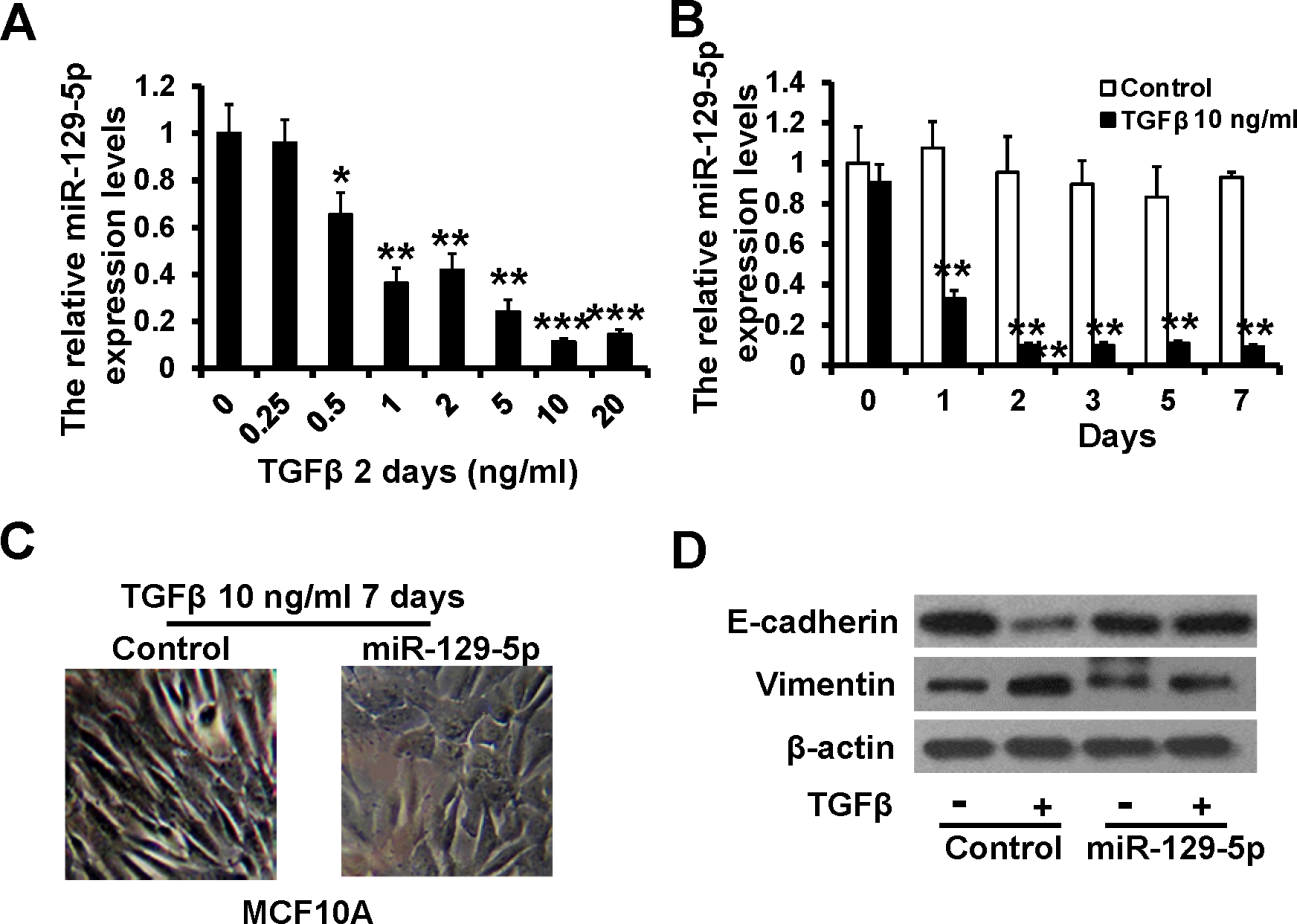
miR-129-5p inhibits TGF-β-induced EMT **A** and **B.** RT-qPCR analysis of the relative miR-129-5p expression levels in MCF10A cells treated with TGF-β1 at indicated concentrations (A) and time (B) compared to the control. **C.** Morphology of MCF10A cells with mimic-Control and miR-129-5p-mimic after 7-day treatment of TGF-β1 (10 ng/ml) in MCF10A cells. **D.** Immunoblotting of E-cadherin and Vimentin in MCF10A cells as treated in C. (***P < 0.001, **P < 0.01, *P < 0.05).

TGF-β signaling induces EMT in MCF10A epithelial cell lines [24]. Here, we showed that miR-129-5p was suppressed in a dose- and time-dependent manner upon the addition of TGF-β to the cell culture medium (Figure 2A and 2B). To investigate whether down-regulation of miR-129-5p is required for TGF-β-induced EMT, we over-expressed miR-129-5p in MCF10A cells and examined their responses to TGF-β1 treatments. MCF10A cells transfected with mimic-Control (MCF10A-mimicControl) displayed an elongated fibroblast-like morphology, whereas miR-129-5p-mimic-transfected cells (MCF10A-miR-129-5pmimic) retained their cobblestone-like morphology after TGF-β1 treatments (Figure 2C). We than examined both epithelial and mesenchymal markers by immunoblotting. Consistent with morphologic changes, E-cadherin expression was decreased, while vimentin expression was increased in MCF10A-mimicControl cells after TGF-β1 treatments, but neither changed expression in MCF10A-miR-129-5pmimic cells (Figure 2D). These results suggest that miR-129-5p is involved in TGF-β-induced EMT.

### miR-129-5p suppresses EMT in human breast cancer cells

**Figure 3:**
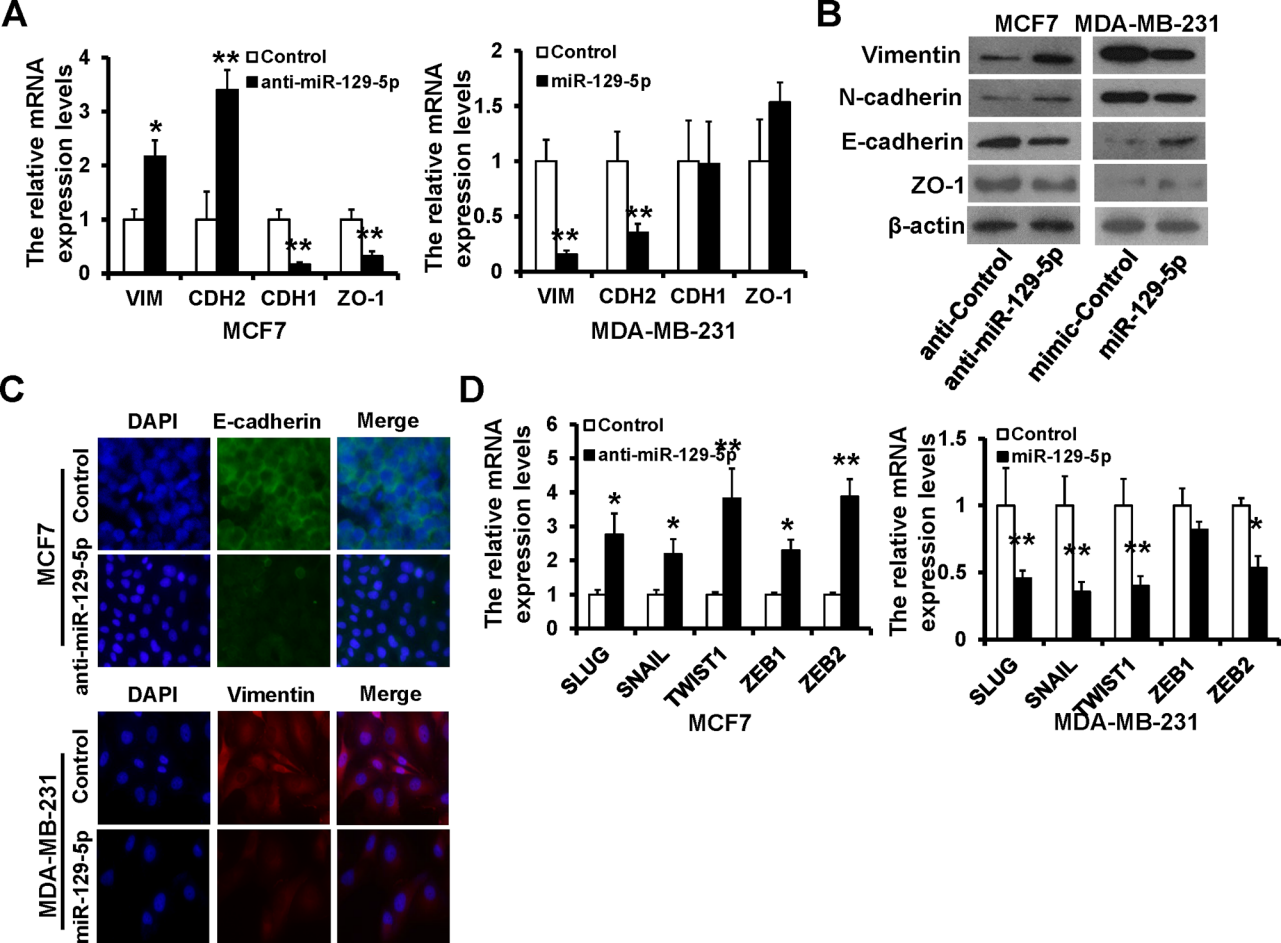
miR-129-5p depletion is linked to the EMT-like phenotype **A** and **B.** RT-qPCR (A) and immunoblotting (B) analyses of expression of the mesenchymal markers Vimentin and N-cadherin, and epithelial markers E-cadherin and ZO-1 in the miR-129-5p-depleted MCF7, miR-129-5p-overexpressed MDA-MB-231, or appropriate control cells. **C.** Immunofluorescence staining for the epithelial and mesenchymal markers. **D.** RT-qPCR analysis of the mRNA levels of EMT-inducers. (**P < 0.01, *P < 0.05).

To further examine the effect of miR-129-5p expression in breast cancer EMT, we measured expression of epithelial and mesenchymal markers by RT-qPCR and immunoblotting. miR-129-5p-depleted MCF7 cells exhibited a significant up-regulation of vimentin and N-cadherin, while epithelial markers E-cadherin and ZO-1 were dramatically down-regulated (Figure 3A, left and Figure 3B, left). In contrast, vimentin and N-cadherin were down-regulated, while E-cadherin and ZO-1 were up-regulated in miR-129-5p-overexpressing MDA-MB-231 cells (Figure 3A, right and 3B, right). E-cadherin protein expression was decreased in miR-129-5p-depleted MCF7 cells whereas vimentin protein expression was decreased in miR-129-5p-overexpressed MDA-MB-231 cells compared to controls (Figure 3C). To probe the interactions between miR-129-5p and EMT-inducing transcription factors, we examined mRNA levels of known EMT inducers Slug, Snail, Twist1, ZEB1, and ZEB2. Expression of all five transcription factors increased in response to miR-129-5p depletion in MCF7 cells (Figure 3D, left), while all but ZEB1 had decreased expression in response to miR-129-5p over-expression,in MDA-MB-231 cells (Figure 3D right). These results suggest that miR-129-5p is a suppressor of breast cancer EMT.

### Twist1 is a direct target of miR-129-5p

**Figure 4:**
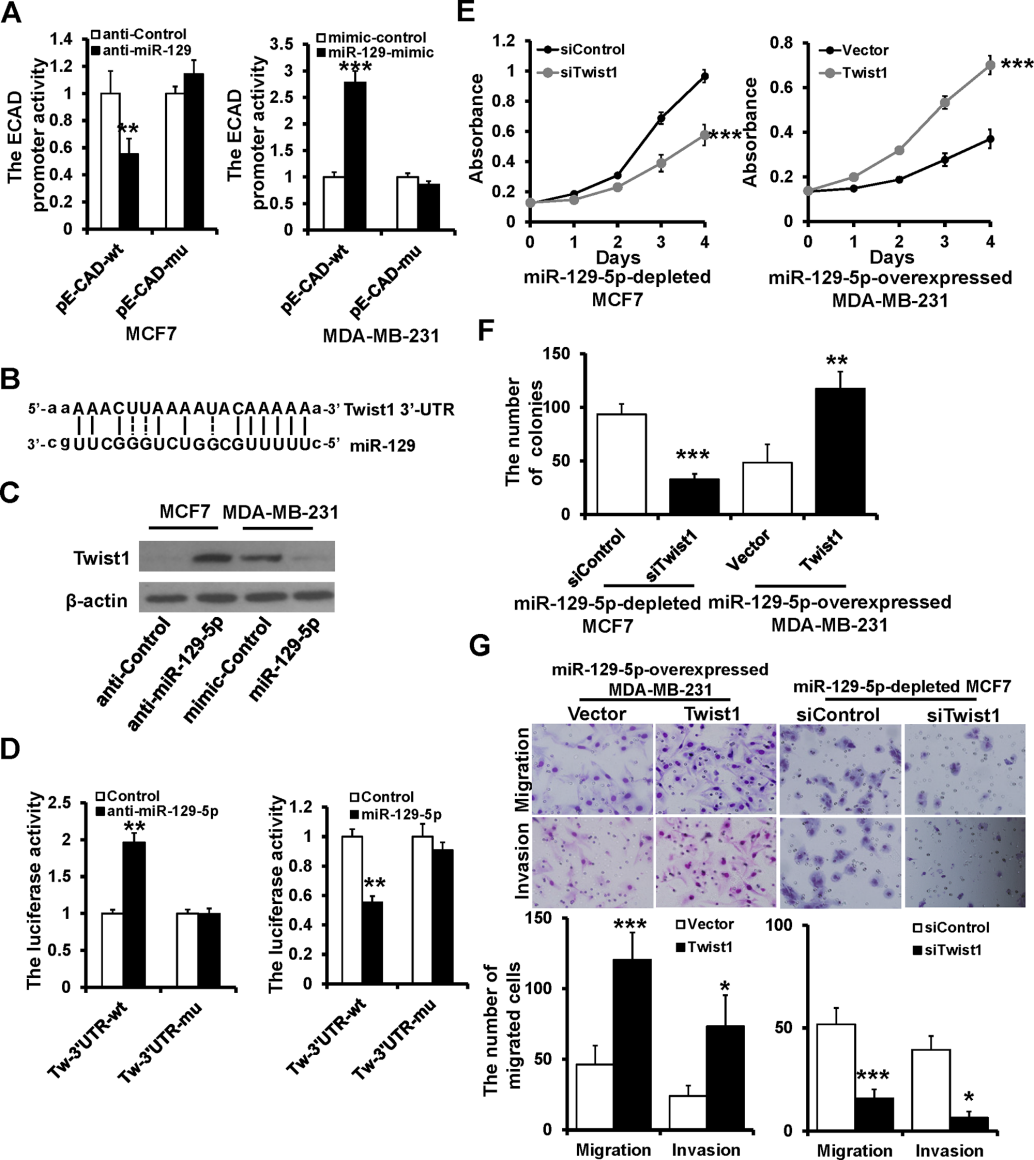
Twist1 is a direct target of miR-129-5p **A.** Dual luciferase reporter system analysis of E-cadherin promoter activity. The wild-type E-cadherin promoter (pE-CAD-wt) or the E-box-mutated E-cadherin promoter (p-E-CAD-mu) transfected into MCF7 and MDA-MB-231 cells with anti-miR-129-5p, miR-129-5p-mimic, or appropriate controls. **B.** The predicted binding of miR-129-5p with Twist1 3′UTR. **C.** Immunoblotting analysis of Twist1 expression in MCF7 treated with anti-miR-129-5p or MDA-MB-231 cells treated withmiR-129-5p-mimic. **D.** Dual luciferase reporter system analysis of was performed to validate miR-129-5p target Twist1. A3′UTR fragment containing the predicted miR-129-5p targeting sites of Twist1 was fused downstream of the Luc gene in pGL3-control plasmid (Tw-3′UTR-wt). A miR-129-5p mutated binding site was also constructed (Tw-3′UTR-mu). **E, F** and **G.** Twist1 rescued the repressive effect of miR-129-5p on breast cancer cell proliferation, migration and invasion by MTT (E), colony formation (F), and transwell (G) assay. (***P < 0.001, **P < 0.01, *P < 0.05).

Loss of E-cadherin is a critical event in EMT. We next used a luciferase reporter assay to investigate whether miR-129-5p transcriptionally regulates E-cadherin expression. Relative luciferase activity of E-cadherin was decreased in MCF7 cells transiently co-transfected with the E-cadherin promoter (pE-CAD-wt) and miR-129-5p inhibitor or control (Figure 4A, left). Conversely, luciferase activity was increased in MDA-MB-231 cells transiently co-transfected with pE-CAD-wt and miR-129-5p mimic or control (Figure 4A, right). To further explore the interaction of miR-129-5p with the E-cadherin promoter, we co-transfected an E-box mutated E-cadherin promoter (pE-CAD-mu) with miR-129-5p inhibitor/mimic or control. Relative luciferase activity was not changed in either MCF7 and MCA-MB-231 cells (Figure 4A), indicating that miR-129-5p does not directly regulate E-cadherin transcription.

Twist1 was predicted as a potential target gene of miR-129-5p based on miR-target analysis (http://www. microrna.org). The predicted binding of miR-129-5p with the Twist1 3′UTR is shown in Figure 4B. To demonstrate that miR-129-5p regulates the expression of Twist1, miR-129-5p inhibitor and mimic were transfected into MCF7 and MDA-MB-231 cells, respectively. Twist1 protein expression increased when MCF7 cells were treated with miR-129-5p inhibitor and decreased when MDA-MB-231 cells were treated with miR-129-5p mimic (Figure 4C). To examine whether miR-129-5p directly interacts with the predicted 3′UTR of Twist1, the 3′UTR of human Twist1 was cloned downstream of the LUC coding gene of pGL3-control (Tw-3′UTR-wt) and co-transfected with miR-129-5p inhibitor or mimic into MCF7 or MDA-MB-231 cells, respectively. Luciferase activity was significantly increased in miR-129-5p depleted MCF7 cells (Figure 4D left), whereas it was decreased in miR-129-5p over-expressing MDA-MB-231 cells compared with controls (Figure 4D right). In addition, site-directed mutagenesis of the seed region abolished the inhibitory effect of miR-129-5p on luciferase activity in both MCF7 and MDA-MB-231 cells (Figure 4D).

To ascertain whether miR-129-5p regulates breast cancer progression through its interaction with Twist1, we performed a rescue experiment. Knockdown or overexpression of Twist1 rescued the effects of miR-129-5p depletion or overexpression on breast cancer proliferation (Figure 4E and 4F), migration, and invasion (Figure 4G) in MCF7 and MDA-MB-231 cells. These data indicate that miR-129-5p targets Twist1, resulting in the negative regulation of breast cancer proliferation, migration, and invasion.

### miR-129-5p is a transcriptional target of Twist1 and Snail

**Figure 5:**
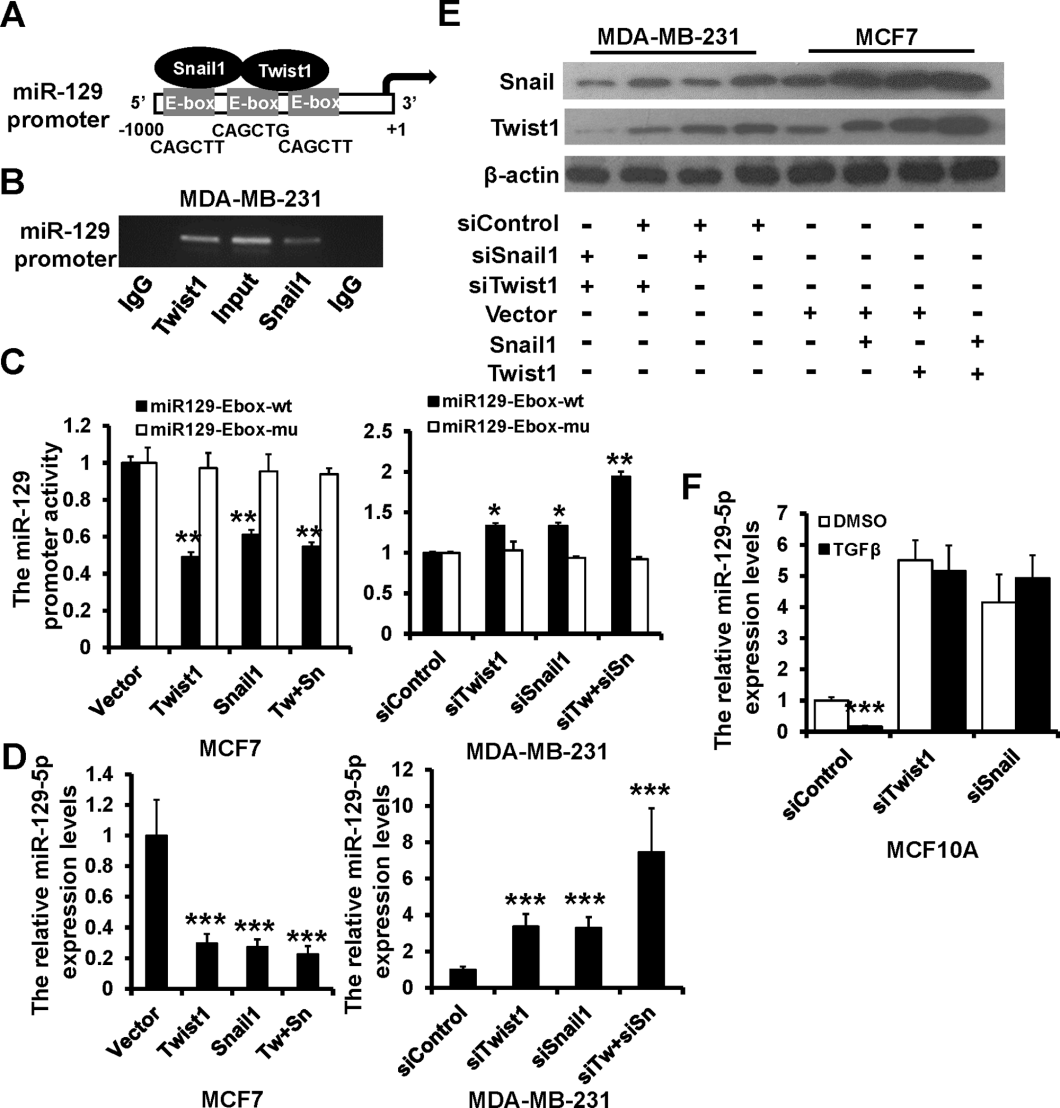
Snail and Twist1 transcriptional suppresses miR-129-5p expression **A.** Schematic representation of miR-129 promoter region with three E-boxes. **B.** PCR amplification of the anti-Twist1 or Snail-enriched miR-129 promoter fragments in MDA-MB-231 cells using a ChIP assay. **C.** Dual luciferase reporter system analysis of miR-129 promoter activity. The wild-type miR-129 promoter (miR129-Ebox-wt) or the E-box-mutated miR-129 promoter (miR129-Ebox-mu) transfected into MCF7 and MDA-MB-231 cells with Twist1 and/or Snail mammalian expression plasmids, siRNAs, or appropriate controls. **D.** RT-qPCR analysis of miR-129-5p expression in MCF7 and MDA-MB-231 cells as treated in C. **E.** Immunoblotting analysis of Twist1 and Snail expression in MCF7 and MDA-MB-231 cells as treated in C. **F.** RT-qPCR analysis of the relative miR-129-5p expression levels in Twist1 or Snail-depleted MCF10A cells treated with TGF-β1 (10 ng/ml, 2 days) compared to the control. (***P < 0.001, **P < 0.01, *P < 0.05).

Analysis of the miR-129 promoter revealed three E-box motifs (™300 to +1 bp; Figure 5A). To investigate the binding of Twist1 and Snail to the miR-129 promoter, we performed a ChIP assay in MDA-MB-231 cells. The miR-129 promoter fragment was enriched with both anti-Twist1 and anti-Snail antibodies but not by IgG control (Figure 5B), suggesting that both Twist1 and Snail can bind to the miR-129 promoter region. Next, we investigated whether Twist1 and Snail regulate the transcriptional activation of miR-129 using a dual-luciferase reporter assay. The miR129-Ebox-wt plasmid, which contained the fragment of miR-129 promoter, was transiently co-transfected with Twist1 and/or Snail plasmids into MCF7 cells, or with Twist1 and/or Snail siRNAs and their controls into MDA-MB-231 cells. miR-129 promoter activity was decreased in Twist1and/or Snail-overexpressed MCF7 cells, whereas activity was increased in Twist1 and/or Snail-depleted MDA-MB-231 cells compared to those of controls (Figure 5C). We next co-transfected miR-129-5p, an Ebox-mutated luciferase report plasmid (miR129-Ebox-mu), with Twist1 and/or Snail plasmids into MCF7 cells, or with Twist1 and/or Snail siRNAs into MDA-MB-231 cells. The miR129-Ebox-mu was no longer responsive to the presence of Twist1 or Snail (Figure 5C).

We performed RT-qPCR to confirm the effect of Twist1/Snail on miR-129-5p expression. miR-129-5p was down-regulated in Twist1 and/or Snail-overexpressed MCF7 cells, whereas it was up-regulated in Twist1 and/or Snail-depleted MDA-MB-231 cells (Figure 5D). Consistent with previous studies, we demonstrated the existence of a Twist1-Snail positive feedback loop by immunoblotting (Figure 5E). miR-129-5p expression was decreased in TGF-β1 treated MCF10A cells (Figure 2A and 2B); however, miR-129-5p expression was not altered in Twist1- or Snail-ablated MCF10A cells after TGF-β1 treatment (Figure 5F). Together, these results demonstrate that both Twist1 and Snail negatively regulate miR-129-5p expression.

### miR-129-5p is down-regulated in breast cancer and correlates with patients’ prognosis

**Figure 6:**
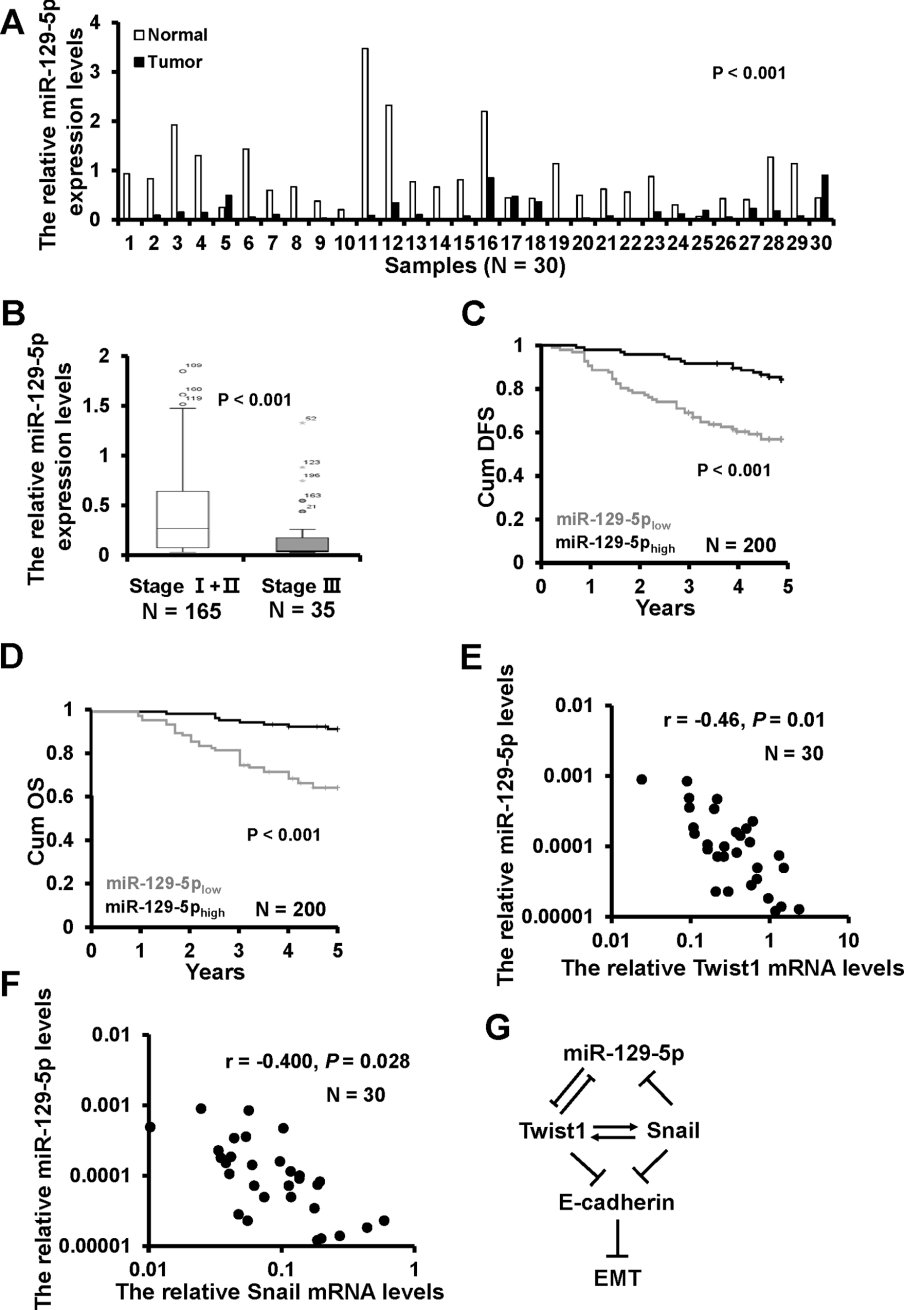
miR-129-5p down-regulation in breast cancer samples with shorter patient DFS **A.** The miR-129-5p expression in 30 cases of breast cancer tissues (T) and the paired normal breast (N) **B.** The box plot depicts miR-129-5p expression levels by RT-qPCR in 200 cases of breast cancer samples classified according clinical stage. **C.** Kaplan-Meier survival analysis of DFS according to the miR-129-5p expression levels. **D.** Kaplan-Meier survival analysis of OS according to the miR-129-5p expression levels. **E.** The relationship between miR-129-5p and Twist1 mRNA expression in 30 cases of breast cancer samples by RT-qPCR. **F.** The relationship between miR-129-5p and Snail mRNA expression in 30 cases of breast cancer samples by RT-qPCR. **G.** A model of miR-129-5p's regulation of EMT in breast cancer.

To examine whether the biological effects of miR-129-5p on breast cancer progression were clinically relevant, we assessed its expression in 30 cases of paired normal and breast cancer tissues. miR-129-5p expression was down-regulated in 26/30 collected breast cancer tissues as compared with paired adjacent normal breast tissues (Figure 6A). Furthermore, miR-129-5p expression levels were lower in advanced tumor stage III than in tumor stages I/II (Figure 6B). We then subdivided the tumors into two groups according to the miR-129-5p median expression and analyzed the relationship between miR-129-5p expression and clinicopathologic features known to affect patient outcome, including patient age, menopausal status, tumor size, clinical stage, histological grade, lymph node status, and ER/PR/Her2 status. As shown in Table 1, miR-129-5p expression levels were correlated with clinical stage (*P* < 0.001), histological grade (*P* < 0.001), lymph node status (*P* < 0.001) and PR status (*P* = 0.016). A Kaplan-Meier survival analysis revealed that patients in miR-129-5p_high_ group had a more favorable outcome than those in the miR-129-5p_low_ group (Figure 6C).

**Table 1:** Association between miR-129-5p expression levels and clinicopathological factors

Clinicopathological Factors	miR-129-5p_High_ (*N* = 100)	miR-129-5p_Low_ (*N* = 100)	*P*
**Age (year)**			
<55	69	61	0.299
> =55	31	39	
**Menopausal status**			
Pre-	51	51	1.000
Post-	49	49	
**Tumor size (cm)**			0.064
≤2	29	17	
>2	71	83	
**Clinical stage**			
I+II	94	71	<0.001
III	6	29	
**Histological grade**			
I+II	94	74	<0.001
III	6	26	
**Lymph node status**			
Negative	51	24	<0.001
Positive	49	76	
**ER status**			
Positive	62	55	0.315
Negative	38	45	
**PR status**			
Positive	59	41	0.016
Negative	41	59	
**HER-2 status**			
Negative	65	71	0.389
Positive	35	29	

Multivariate analysis was performed to determine the prognostic value of miR-129-5p expression using the Cox proportional hazard model. The risk variables examined included miR-129-5p expression level, as well as the factors examined in correlation analysis, above. In the univariate analysis, low miR-129-5p expression level (*P* < 0.001), lymph node status (*P* = 0.005), Clinical stage (*P* < 0.001), and histological grade (*P* = 0.022) were significantly associated with survival (Table 2). In the final multivariate Cox regression model, low miR-129-5p expression (*P* = 0.012) and advanced clinical stage (*P* = 0.008) were associated with a poor survival prognosis independent of other clinical covariates (Table 2). To address whether the expression of miR-129-5p is associated with its targets or regulators, both Twist1 and Snail mRNA expression were examined in 30 cases of primary breast cancer tissues by RT-qPCR. There was a significant inverse correlation between miR-129-5p and Twist1 (Figure 6E) or Snail (Figure 6F) expression inav breast cancer tissues. These results indicate that lower miR-129-5p expression is associated with breast cancer progression and a worse prognosis.

**Table 2:** Univariate and multivariate analysis of various clinicopathological variables for relapse and metastasis risk

Variable	Comparison	HR (95% CI)	*P* - value
**Univariate**			
Tumor size	> 2 *vs.* ≤ 2	1.518 (0.766–3.009)	0.231
Lymph node status	Pos. *vs.* Neg.	2.497 (1.318–4.728)	0.005
Clinical stage	III *vs*. I–II	3.880 (2.253–6.681)	<0.001
Histological grade	III *vs.* I–II	2.025 (1.106–3.708)	0.022
miR-129 expression	miR-129-5p_low_ *vs.* miR-129-5p_high_	3.784 (2.064–6.937)	<0.001
**Multivariate**			
Tumor size	> 2 *vs.* ≤ 2	1.208 (0.598–2.439)	0.598
Lymph node status	Pos. *vs.* Neg.	1.583 (0.801–3.130)	0.186
Clinical stage	III *vs.* I–II	2.286 (1.246–4.194)	0.008
Histological grade	III *vs.* I–II	1.396 (0.744–2.617)	0.299
miR-129-5p expression	miR-129-5p_low_ *vs.* miR-129-5p_high_	2.394 (1.210–4.738)	0.012

## DISCUSSION

miRNAs are emerging as a new class of tumor suppressor genes or oncogenes in cancer [25, 26] and, in the future, they may serve as new biomarkers to predict clinical outcomes [27]. In this study, we provide the first evidence that miR-129-5p plays a role in breast carcinogenesis [7]. Its expression is down-regulated in breast cancer tissues, with lower levels in advanced tumor stages. miR-129-5p down-regulation is associated with a poor prognosis. The negative relationship of miR-129-5p expression levels with proliferation and motility supports the notion that it acts as a tumor suppressor miRNA. miR-129-5p was first found to be down-regulated in undifferentiated gastric cancer tissues [28] where it inhibits gastric cancer proliferation [13]. Later it was found to inhibit growth and induce cell death in hepatocellular carcinoma, bladder carcinoma, colorectal carcinoma, esophageal carcinoma, and laryngeal squamous cell carcinoma [14, 18, 21, 29]. Conversely, it is up-regulated in esophageal squamous cell carcinoma [20]. These conflicting data suggest that miR-129-5p may have different functions depending on the cell context and cancer type. Our data suggest that its down-regulation is associated with more aggressive tumors and a poorer prognosis in breast cancer. Promoter hypermethylation down-regulates tumor suppressive miRNAs and correlates with carcinogenesis. Hypermethylation of the CpG island upstream of miR-129 has been observed in several types of cancer, however, the methylation levels of miR-129 in breast cancer is still unknown. Thus, the epigenetic regulation of miR-129 in breast cancer cells should be examined in future studies.

EMT enables epithelial cells to acquire an invasive mesenchymal phenotype and is a key mechanism for the initial step of metastasis [30, 31]. Recently, several miRNAs, such as the miR-200 [32] and miR-34 [33] families, have been found to participate in the regulation of EMT. Our data demonstrates that over-expression of miR-129-5p prevents TGF-β-induced EMT and down-regulation of miR-129-5p is required for EMT initiation and maintenance in breast epithelial cells. In addition, it is sufficient to reverse the mesenchymal characteristics and decrease the invasiveness of mesenchymal cells. In contrast, inhibition of miR-129-5p in epithelial cells triggered EMT, resulting in a more invasive phenotype.

miR-129-5p suppressed EMT in human breast cancer by directly targeting Twist1. Twist1 promotes the EMT process [34, 35], and elevated Twist1 expression is also correlated with tumor proliferation, invasion, and metastasis in a variety of solid tumors, including breast cancer [36], lung cancer [37], prostate cancer [38], and oral cancer [39]. miR-300 and miR-720 inhibit EMT and metastasis by targeting Twist1 expression [40, 41]. Similarly, we show that Twist1 is a direct target of miR-129-5p. Moreover, there is a significant inverse correlation between miR-129-5p and Twist1 expression levels in breast cancer tissues, suggesting a functional interaction of miR-129-5p and Twist1.

Both Twist1 and Snail are over-expressed in various human solid tumors and enhance proliferation, invasion, and metastasis by induction of EMT [34, 42, 43]. They bind to the E-box motif (canonical, 5′-CANNTG-3′ and non-canonical, 5′-CANNTT-3′) in target gene promoters and suppress the expression of target genes [7, 43]. Our work indicates that both Twist1 and Snail bind to the miR-129-5p promoter region and suppress its expression. As Twist1 and Snail play an essential role in EMT as well as breast cancer metastasis, our data establish a mechanistic link between miR-129-5p, Twist1, Snail, in EMT and breast cancer metastasis.

## MATERIALS AND METHODS

### Cell culture

MCF10A, MCF7, T47D, BT474, BT549, MDA-MB-468, MDA-MB-231 cell lines were obtained from the Cell Bank of the Chinese Academy of Sciences (Shanghai, China). MCF10A was cultured in DMEM/F12 supplemented with 5% horse serum (Life Technologies), 10 μg/mL insulin (Sigma-Aldrich, St Louis, MO, USA), 0.5 μg/mL hydrocortisone (Sigma), 20 ng/mL EGF (R&D Systems), and 100 ng/mL cholera toxin (Sigma). BT549 cells were cultured in RPMI-1640 medium supplemented with 10% FBS and 0.023 IU/mL insulin. BT474 cells were cultured in RPMI-1640 medium supplemented with 15% FBS and 0.1 IU/mL insulin. MDA-MB-231 cells were cultured in RPMI-1640 medium supplemented with 10% FBS. T47D and MCF7 cells were cultured in DMEM supplemented with 10% FBS. The cells were incubated at 37°C in a 5% CO_2_ atmosphere. MDA-MB-468 cells were cultured in Leibovitz's L-15 medium supplemented with 10% FBS without CO_2_ at 37°C. All cell lines were cultured with 100 μg/mL streptomycin and 100 units/mL penicillin. The cells were expanded immediately and multiple aliquots were cryopreserved. Cells were used within 6 months of resuscitation.

### Clinical samples

Breast cancer specimens were obtained from Tianjin Medical University Cancer Institute and Hospital. A total of 200 paraffin-embedded specimens and 30 primary breast cancer tissue and the paired adjacent normal breast tissue specimens were included in this study. All tumors were from patients with a newly diagnosed breast cancer who had received no therapy before sample collection. After radical prostatectomy, the primary breast cancer tissue and the adjacent normal tissues were flash-frozen in liquid nitrogen and stored at −80°C. This study was approved by the Institutional Review Board of the Tianjin Medical University Cancer Institute and Hospital and written consent was obtained from all participants.

### Antibodies and reagents

Antibodies against N-cadherin, E-cadherin, Vimentin, ZO-1 (Santa Cruz Biotechnology, Santa Cruz, CA, USA), Twist1, Snail (Abcam, Cambridge, MA, USA) and β-actin (Cell Signaling Technology, Beverly, MA, USA) were used. Recombinant human TGFβ1 was purchased from R&D Systems (Redmond, WA, USA).

### Plasmids, miRNAs, and small interfering RNAs

The ORF of human Twist1 and Snail genes were amplified by PCR in 293FT cell line, the amplified fragments were subcloned into the pcDNA3.1/myc-hisA vector. The pE-CAD-wt and pE-CAD-mu were a gift from Kumiko UiTei. The Twist1 3′-UTR containing miR-129-5p binding site or miR-129-5p binding site mutated fragments were cloned into pGL3-Control vector (Promega, Madison, WI, USA; Tw-3′UTR-wt and Tw-3′UTR-mu). The miR-129 promoter region (−300 to +1) and the E-box mutated fragments were cloned into pGL3-Basic vector (Promega; miR-129-Ebox-wt and miR-129-Ebox-mu). The miR-129-5p mimic and inhibitor were purchased from RiboBio (Shanghai, China). The Twist1 gene-specific short interfering (siRNA), Snail siRNA, and non-specific control siRNA were also purchased from RiboBio.

### Transfection and luciferase assay

miR-129-5p mimic (miR-129-5p-mimic), miR-129-5p inhibitor (anti-miR-129-5p), or the appropriate scrambled controls (mimic-control and anti-control) and the siRNAs were transfected into different cell lines using lipofectamine RNAiMAX reagent (Life Technologies) according to the manufacturer's recommendations. The mammalian expression plasmids were transfected into different cells using lipofectamine 3000 reagent (Life Technologies) according to the manufacturer's recommendations.

Luciferase assays were carried out on extracts from different breast cancer cells co-transfected for 24/48 hours with the corresponding plasmids, miRNAs, or siRNAs by using a dual luciferase assay kit (Promega) according to the manufacturer's recommendations. The results were normalized against *Renilla* luciferase activity. All transfections were performed in triplicate.

### Proliferation, migration and invasion assays

Both MTT and plate colony formation assays were used to evaluate the ability of cell proliferation. For MTT assay, 24 h after transfection, 5 × 10^3^ cells were seeded in 96-well plates per well. After incubation for indicated time, the cells were incubated with 10 μl MTT (0.5 mg/ml; Sigma-Aldrich) at 37°C for 4 h. Then, the medium was removed, and precipitated Formosan was dissolved in 150 μl DMSO. The absorbance at 570 nm was detected using a micro-plate auto-reader (Bio-Rad, Richmond, CA, USA). For the plate colony formation assay, 24 h after transfection, 500 cells were seeded in 6-well plates per well. After about 3 weeks, the colonies obtained were washed with PBS and fixed with 10% formalin for 15 min at room temperature and then washed with PBS followed by staining with hematoxylin. The colonies were counted and compared with control cells.

The invasion and migration abilities of breast cancer cells *in vitro* were evaluated by Matrigel coated Transwell and Transwell inserts (BD Biosciences, San Diego, CA, USA), respectively. Briefly, 5 × 10^4^ cells in 500 μl serum-free medium were added to the upper chamber, and medium containing 20% FBS was added into the lower chamber. 24 h later, the migrant cells that had attached to the lower, surface were fixed with 20% methanol and stained for 20 min with crystal violet. The membranes were then carved and embedded under cover slips with the cells on the top. The number of migrating cells was counted under a microscope in five predetermined fields.

### RNA extraction and reverse transcription quantitative PCR

Total RNA of cultured cells, surgically resected fresh breast tissues, and formalin-fixed paraffin-embedded clinical specimens were extracted using mirVana PARIS kit (Life Technologies) according to the manufacturer's instruction. For mRNA detection, reverse transcription was performed using a First Strand cDNA Synthesis kit (TakaRa, Dalian, China), according to the manufacturer's instruction. The real-time quantitative PCR was performed using GoTaq qPCR Master Mix (Promega) on a Bio-Rad iQ5 Optical System (Bio-Rad, Hercules, CA, USA). β-actin was used as an internal control. For miRNA detection, miRNA was reverse transcribed with TaqMan MicroRNA Reverse Transcription kit and real-time quantitative PCR was performed using TaqMan miR-129-5p and u6 RNA (used as a normalizer) assays (Life Technologies) following the manufacturer's instructions.

### Immunoblotting and immunofluorescence

For immunoblotting assays, cells were lysed in protein lysis buffer (20 nm Tris-HCl pH 7.4, 5 mM EDTA, 1% Trition X-100, 150 mM NaCl, 1% DTT) containing a protease inhibitor cocktail tablet (Roche Molecular Biochemicals, Indianapolis, IN, USA). Protein lysates were resolved by SDS-PAGE, transferred to PVDF membranes (Millipore, Bedford, MA, USA), detected with primary antibody overnight at 4°C, and then incubated with HRP-conjugated secondary antibodies. The blots were visualized with ECL reagent (Millipore).

For immunofluorescence assays, cell were seeded onto glass coverslips in 24-well plates, washed with PBS, fixed in 4% formaldehyde solution for 30 min and then permeabilized with 0.2% Triton X-100/PBS for 15 min. Cells were blocked with 2% BSA in PBS for 30 min. Coverslips were incubated with primary antibodies overnight at 4°C, followed by incubation with FITC-/TRITC-conjugated secondary antibodies for 1 h at room temperature, and then stained with DAPI. Finally, coverslips were observed under a fluorescence microscope.

### Chromatin immunoprecipitation

The Chromatin immunoprecipitation (ChIP) assay was carried out by using a ChIP assay kit (Upstate, Lake Placid, NY, USA) according to the manufacturer's recommendations. Briefly, approximately 1 × 10^7^ cells were crosslinked with 1% formaldehyde and resuspended, lysed in lysis buffer, then sonicated to shear the genomic DNA to a range of 500 bp to 2000 bp. The chromatin fraction was immunoprecipitated by using an anti-Twist1 or anti-Snail antibody. The chromosomal DNA was purified by using a MinElute PCR purification Kit (Qiagen, Hilden, Germany) according to the manufacture's instruction. PCR was carried out using primers specific for the Twist1 or Snail binding region in the human mir-129 promoter (forward 5′-GGCGCAGGATGCAGGAGAT-3′ and reverse 5′-GAGGGAGAGCCAGGACCTA-3′). The PCR products were separated on a 2% agarose gel containing ethidium bromide.

### Statistical analysis

Data are presented as mean ± standard deviation. Student's *t*-test was used to compare the differences between the experimental group and control group. The relationship between miR-129-5p expression and various clinicopathological variables was analyzed by the chi-square test. Kaplan-Meier survival curves and the log-rank test were used to evaluate the breast cancer patients with different miR-129-5p expression. Multivariate survival analysis was performed by a backward stepwise Cox proportional hazards regression model. All calculations were performed with the SPSS for Windows statistical software package (SPSS Inc., Chicago, IL, USA). The level of significance was set to *P* < 0.05.

## CONCLUSION

In summary, we find that miR-129-5p suppresses breast cancer development and progression and that down-regulation of miR-129-5p, through a Twist1-Snail negative feedback loop, is required for EMT initiation and maintenance (Figure 6G).
